# Supplementary material for: Discovery and construction of surface kagome electronic states induced by p-d electronic hybridization in Co3Sn2S2
Source: Nat Commun. 2023 Aug 26;14:5230. doi: 10.1038/s41467-023-40942-2 (PMC10460379; doi:10.1038/s41467-023-40942-2)
Supplement: Supplementary file 1 — Supplementary Information [file 41467_2023_40942_MOESM1_ESM.pdf]

Supplementary Figures for

Discovery and construction of surface kagome electronic states induced by *p-d* electronic hybridization in $Co_3Sn_2S_2$

Li Huang[1,2]†, Xianghua Kong[3,4,5]†, Qi Zheng[1,2]†, Yuqing Xing[1,2]†, Hui Chen[1,2], Yan Li[1,2], Zhixin Hu[6], Shiyu Zhu[1,2], Jingsi Qiao[7,4], Yu-Yang Zhang[2], Haixia Cheng[4], Zhihai Cheng[4], Xianggang Qiu[1,2], Enke Liu[1,2], Hechang Lei[4], Xiao Lin[2], Ziqiang Wang[8], Haitao Yang[1,2]*, Wei Ji[4,9]*, Hong-Jun Gao[1,2,10]*

Affiliations:

[1]Beijing National Center for Condensed Matter Physics and Institute of Physics, Chinese Academy of Sciences, Beijing 100190, China

[2]School of Physical Sciences, University of Chinese Academy of Sciences, Beijing 100190, China

[3]College of Physics and Optoelectronic Engineering, Shenzhen University, Shenzhen 518060, China

[4]Beijing Key Laboratory of Optoelectronic Functional Materials & Micro-Nano Devices, Department of Physics, Renmin University of China, Beijing 100872, China

[5]Centre for the Physics of Materials and Department of Physics, McGill University, Montreal QC H3A 2T8, Canada

[6]Center for Joint Quantum Studies and Department of Physics, Institute of Science, Tianjin University, Tianjin 300350, China

[7]MIIT Key Laboratory for Low-Dimensional Quantum Structure and Devices, School of Integrated Circuits and Electronics, Beijing Institute of Technology, Beijing 100081, China

[8]Department of Physics, Boston College, Chestnut Hill, MA, USA

[9]Key Laboratory of Quantum State Construction and Manipulation (Ministry of Education), Renmin University of China, Beijing, 100872, China

[10]Hefei National Laboratory, Hefei, Anhui 230088, China

* Corresponding author. Email: hjgao@iphy.ac.cn, wji@ruc.edu.cn, htyang@iphy.ac.cn

† These authors contributed equally to this work

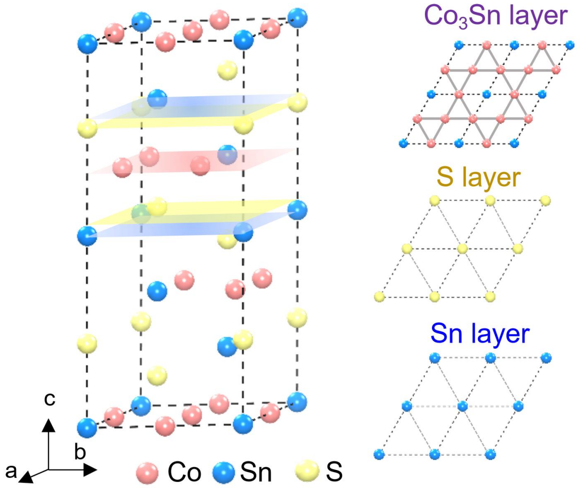

Supplementary Fig. 1 | Atomic structure of $Co_3Sn_2S_2$.

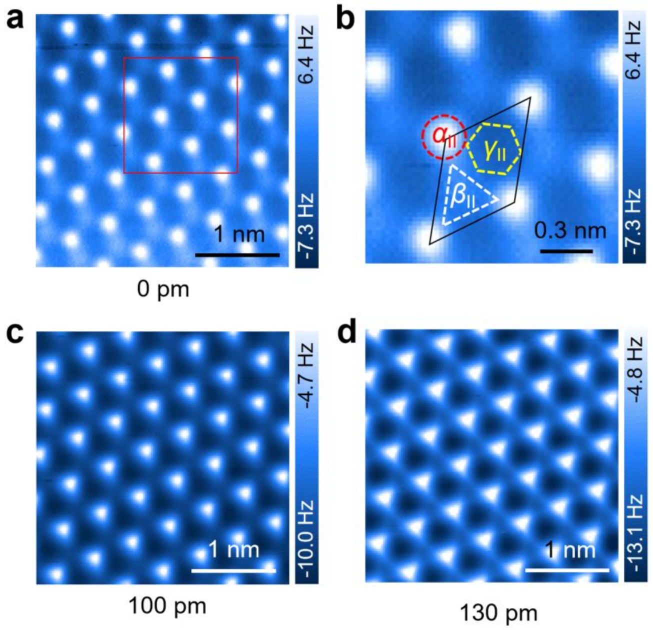

Supplementary Fig. 2 | nc-AFM images at different scanning heights on Type-II surface. (**a**) Constant-height nc-AFM image scanned at the tunneling height of V_s= -4 mV, I_t=10 pA. (**b**) Enlarged from the red square in (**a**) to show the α_{II}, β_{II}, and γ_{II} regions within a supercell. (**c**) and (**d**) Constant-height nc-AFM images at different scanning heights on the Type-II surface. The x and y directions have drifted a little during the scanning time. The number below each image is the scanning height that lowered from a tunneling junction height of V_s= -4 mV, I_t=10 pA. Scanning amplitude = 50 pm.

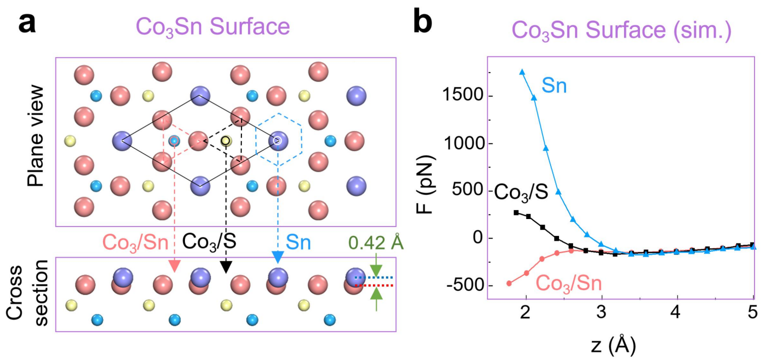

Supplementary Fig. 3 | DFT calculated vertical short-range force spectrum on optimized Co_3Sn surface. (**a**) DFT optimized surface structures of the Co_3Sn surface. (**b**) DFT calculated vertical short-range force spectra on the three typical regions marked by the blue hexagon, black and red triangles in (**a**), plotted with the same color code.

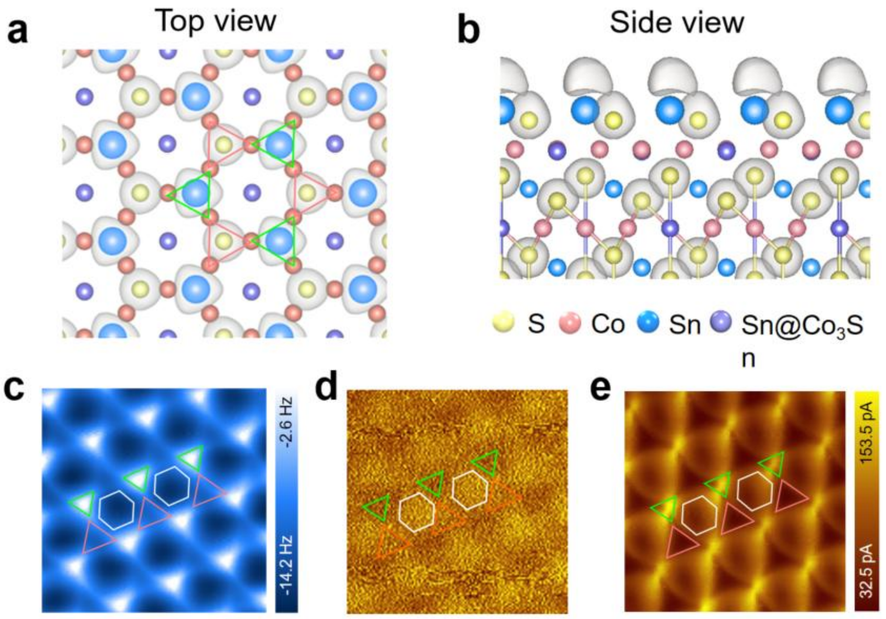

Supplementary Fig. 4 | Localized electronic states of the surface Sn atoms through *p-d* hybridization on Sn surface. (**a**) and (**b**) Top and side view of electron localized function (ELF). Both the surface Sn and subsurface S sites are surrounded by significantly localized electron densities that are crucial for repulsive interactions in nc-AFM imaging. The density above the surface Sn atom is even more localized than that of the S atom, indicating the surface Sn atoms behave like a non-metallic element, which is in opposite to their usually metallic behavior as those bulk Sn atoms. (**c**)-(**e**) Frequency-shift, damping, and current channels of an nc-AFM image. The green and orange triangles and the white hexagon represent the α_{II}, β_{II}, and γ_{II} regions, respectively. The surface Sn atom represents the α_{II} region (green triangle) in the SPM images. Its higher vertical position and the largest localized electron density leads to the strongest repulsion in the frequency-shift image, exhibit no dissipation in the damping image and significant tunneling current in the STM image. As for the embedded Sn (the γ_{II} region), it is highly metallic according to the cross-sectional ELF plot; this allows to dynamically form chemical binds between the embedded Sn and the O atom of the CO tip during scanning. As a result, significant damping signal (white hexagons in **d**) in a nearly hexagonal shape was recorded around the embedded Sn atoms while appreciable tunneling current was obtained in the associated STM image (white hexagons in **e**). The surface S atom (orange triangles, representing region β_{II}) sits at a position in between those two types of Sn atoms in terms of the vertical position, strength of Pauli repulsion and electronic conductance, so that it yields moderate Pauli repulsion, showing blurry triangles in the frequency shift image and nearly insulating feature in the STM image. Such assessment of Sn (surface), S and Sn (embedded) sites to regions α_{II}, β_{II}, and γ_{II} is also consistent with the experimental measurement of force curves.

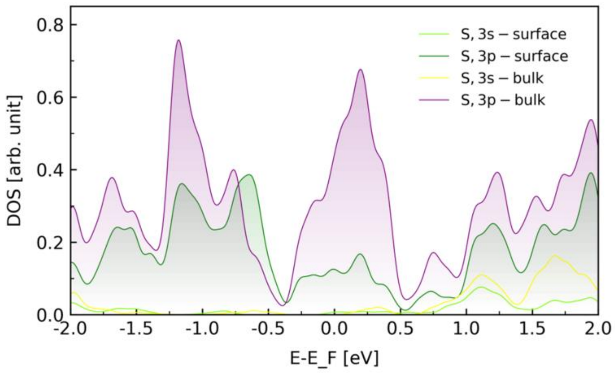

Supplementary Fig. 5 | DFT calculated PLDOS of the orbitals of S atoms in Sn terminated $Co_3Sn_2S_2$. Between the energy range of 0.38-0.50 eV, only the *p* orbitals participate in the hybridization, both for the surface and bulk S atoms.

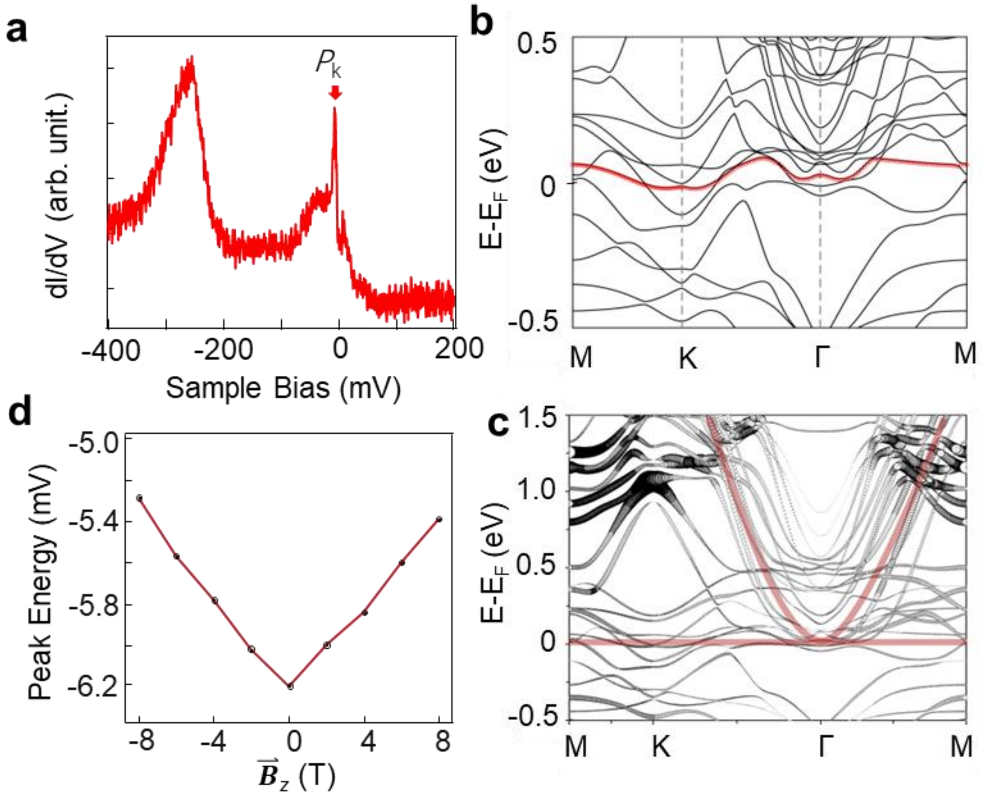

Supplementary Fig. 6 | Electronic properties of the SKES on Sn surface of $Co_3Sn_2S_2$. (**a**) STS on Sn surface. The red arrow marks the flat-band related electronic state P_k residing at around -6 mV. (V_s= -400 mV, I_t = 100 pA, V_{mod} = 0.5 mV). (**b**) DFT calculated electronic band structure with the SOC included, where the flat-band around E_F is highlighted in red. (**c**) The band structures projected on surface *p* orbitals for the Sn surface. A distinct feature of the kagome band structures is resembled, namely the crossing of a flat band and a quadratic band at the Γ point, as depicted with the light-pink straight and the parabolic lines. (**d**) Energy shift of the flat-band peak position as a function of vertical magnetic field, showing the feature of negative orbital magnetism. (V_s= -20 mV, I_t = 500 pA, V_{mod} = 0.1 mV).

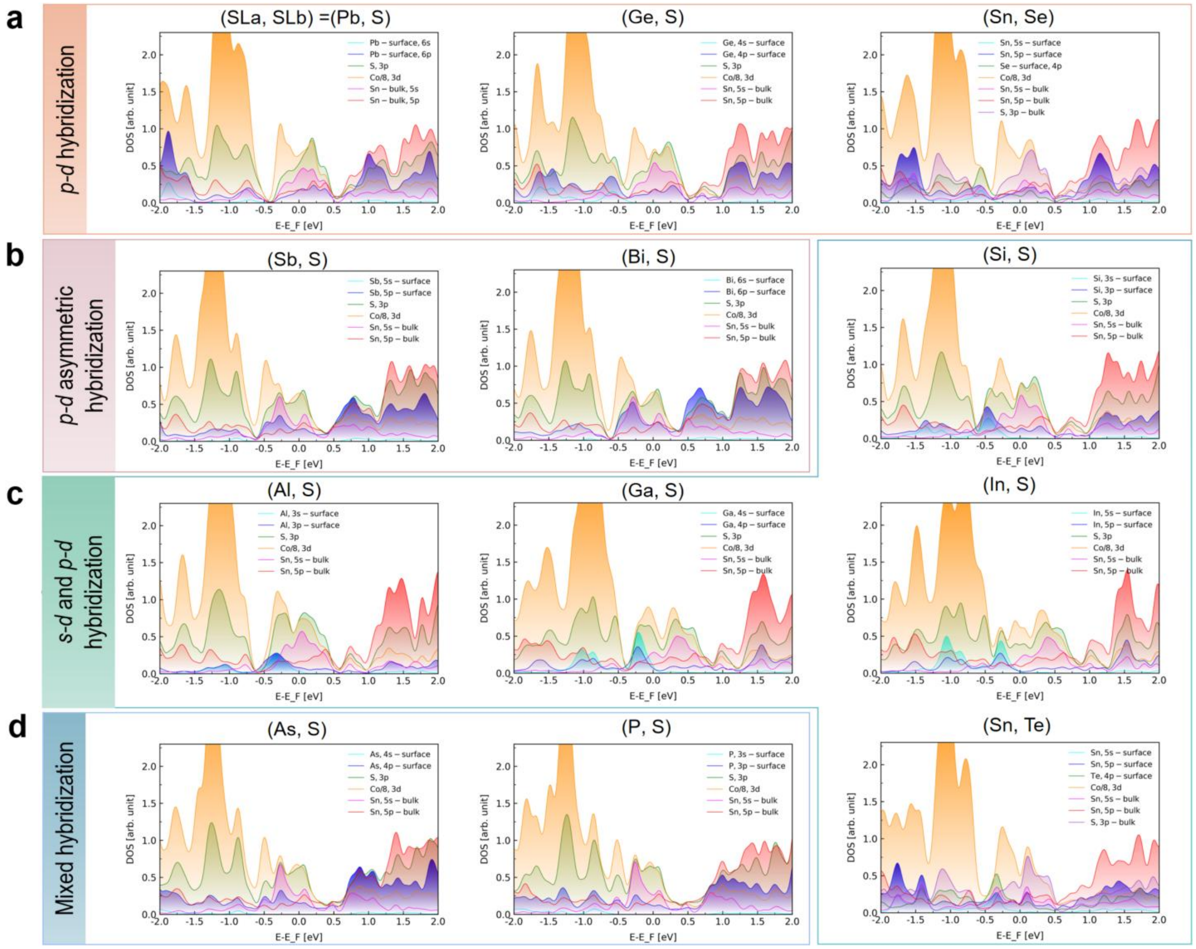

Supplementary Fig. 7 | SKES constructed with different elements in SLa and SLb planes of $Co_3Sn_2S_2$. (**a**) *p-d* hybridized surface electronic states by depositing Pb or Ge elements on S surface or substitute the subsurface S atoms with Se atoms on Sn surface. (**b**) Asymmetric *p-d* hybridized surface electronic states by depositing Sb or Bi elements on S surface. (**c**) *s-d* and *p-d* hybridized surface electronic states by depositing Al, Ga, In or Si elements on S surface, or substitute the subsurface S atoms with Te atoms on Sn surface. (**d**) Mixed hybridized surface electronic states by depositing As or P elements on S surface.
